# Large Scale Analyses and Visualization of Adaptive Amino Acid Changes Projects

**DOI:** 10.1007/s12539-018-0282-7

**Published:** 2018-01-30

**Authors:** Noé Vázquez, Cristina P. Vieira, Bárbara S. R. Amorim, André Torres, Hugo López-Fernández, Florentino Fdez-Riverola, José L. R. Sousa, Miguel Reboiro-Jato, Jorge Vieira

**Affiliations:** 1 ESEI – Escuela Superior de Ingeniería Informática, Edificio Politécnico, Universidade de Vigo, Campus Universitario As Lagoas s/n, 32004 Ourense, Spain; 2 CINBIO - Centro de Investigaciones Biomédicas, University of Vigo, Campus Universitario Lagoas-Marcosende, 36310 Vigo, Spain; 3 Instituto de Investigação e Inovação em Saúde (I3S), Universidade do Porto, Rua Alfredo Allen, 208, 4200-135 Porto, Portugal; 4 Instituto de Biologia Molecular e Celular (IBMC), Rua Alfredo Allen, 208, 4200-135 Porto, Portugal; 5 Instituto Nacional de Engenharia Biomédica (INEB), Rua Alfredo Allen, 208, 4200-135 Porto, Portugal

**Keywords:** ADOPS, Positive selection, B+ database, Open data

## Abstract

When changes at few amino acid sites are the target of selection, adaptive amino acid changes in protein sequences can be identified using maximum-likelihood methods based on models of codon substitution (such as codeml). Although such methods have been employed numerous times using a variety of different organisms, the time needed to collect the data and prepare the input files means that tens or hundreds of coding regions are usually analyzed. Nevertheless, the recent availability of flexible and easy to use computer applications that collect relevant data (such as BDBM) and infer positively selected amino acid sites (such as ADOPS), means that the entire process is easier and quicker than before. However, the lack of a batch option in ADOPS, here reported, still precludes the analysis of hundreds or thousands of sequence files. Given the interest and possibility of running such large-scale projects, we have also developed a database where ADOPS projects can be stored. Therefore, this study also presents the B+ database, which is both a data repository and a convenient interface that looks at the information contained in ADOPS projects without the need to download and unzip the corresponding ADOPS project file. The ADOPS projects available at B+ can also be downloaded, unzipped, and opened using the ADOPS graphical interface. The availability of such a database ensures results repeatability, promotes data reuse with significant savings on the time needed for preparing datasets, and effortlessly allows further exploration of the data contained in ADOPS projects.

## Introduction

Amino acid changes in protein sequences can be adaptive, and when changes at few amino acid sites are the target of selection they can be detected using maximum-likelihood methods based on the models of codon substitution [1–3]. This approach has been applied numerous times to infer positively selected amino acid sites at numerous proteins including, but not limited to: interleukin-3 (IL3), a protein associated with brain volume variation in general human populations [4]; formyl peptide receptors in mammals [5]; scorpion sodium channel toxins [6]; the *Mimulus* plant CENH3 protein [7]; the oyster *Crassostrea gigas* peptidoglycan recognition proteins [8]; host immune response genes [9, 10]; the envelope glycoprotein of dengue viruses [11]; the attachment glycoprotein of respiratory syncytial virus [12]; measles virus hemagglutinin [13]; influenza B virus hemagglutinin [14]; HIV proteins [15]; hemagglutinin-neuraminidase protein of Newcastle disease virus [16]; *Trypanosoma brucei* proteins [17]; the vertebrate skeletal muscle sodium channel protein [18]; the p53 protein [19]; the fruitless protein in *Anastrepha* fruit flies [20]; CC chemokine receptor proteins [21]; or the proteins encoded by plant genes that are involved in gametophytic self-incompatibility specificity determination [22–25]. Recently, it has been argued that pharma and biotech industries can successfully use the knowledge generated by such an approach to deal with real-life problems [26].

Although maximum-likelihood methods based on models of codon substitution have been widely used to infer positively selected amino acid sites, the size of the average project is still relatively small mainly due to the time needed to collect the relevant coding sequences and prepare input files for the different software applications. The recent availability of computer applications such as *Blast DataBase Manager* (BDBM; http://www.sing-group.org/BDBM/) greatly eases the preparation of large datasets. Moreover, the availability of the *Automatic Detection of Positively Selected Sites* (ADOPS) [27] computer application has allowed the automated execution of all the steps needed to infer positively selected amino acid sites, starting from a FASTA file with non-aligned coding sequences; however, the lack of a batch option in this application still means that it is not practical to run thousands of sequence files.

This study will report the implementation of a batch option in the ADOPS software [27] that allows users to easily run large scale analyses involving thousands of genes, using moderate computer resources. Given this improvement, the next logical step is to make ADOPS projects (especially large-scale projects) available to the research community. To that end, we also present B+ (http://bpositive.i3s.up.pt/), a database that has been specifically designed to store and show the information contained in ADOPS project files. Although a database dedicated to positive selection inferences at the codon level has already been published [28], it is dedicated to a specific group of organisms, and the possibility of reusing data is not as easy as with B+ and ADOPS. Both large and small ADOPS datasets can be submitted to B+ (as compressed tar.gz files) along with a description containing the details about how the project was performed. At present, the B+ database hosts the “Closely related Drosophila dataset (2016)”, which provides ADOPS projects for 19,652 Drosophila transcripts, 14.6% of which show signs of positive selection (1200 genes), although curated analyses must now be performed to validate these results.

## ADOPS Batch Mode

Multiple instances of the ADOPS graphical user interface (GUI) can be launched simultaneously, which correspondingly allows for the possibility of multiple parallel processing of ADOPS projects. However, this is only possible if a sufficient amount of memory is available, which in turn is determined by the number of sequences used in the project and the total number of individual projects to be run. A single ADOPS batch project with 50 individual projects, each with an average of 10 sequences per individual project, runs in approximately 1–2 days. This suggests that even with limited computational power it is possible to run about 100 individual projects every 2 days.

**Fig. 1:**
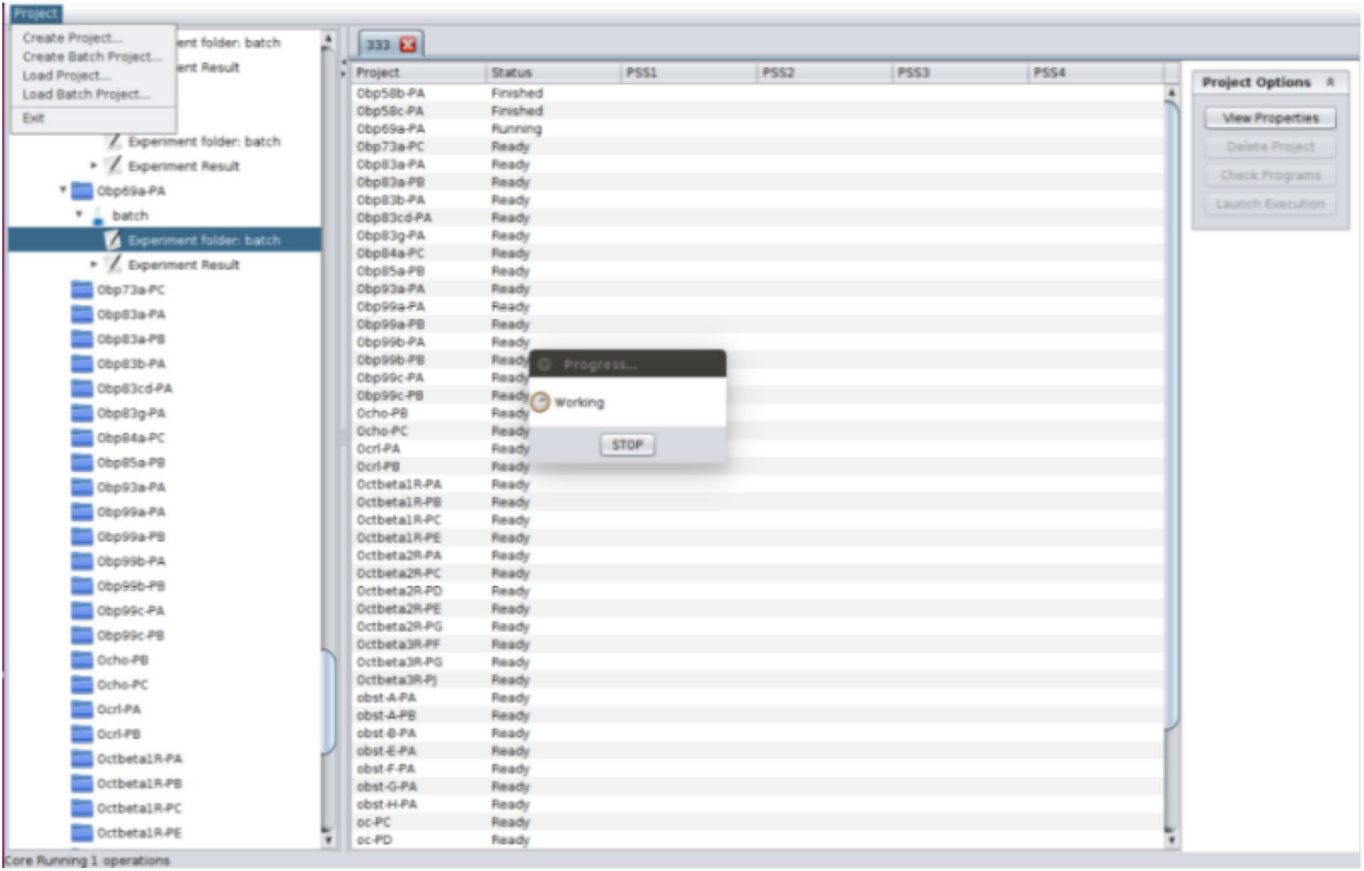
The *‘Create Batch Project’* option

To launch the new batch option implemented in ADOPS, the user launches the GUI and chooses the *‘Create Batch Project’* option under the *‘Project’* menu (Fig. 1). The user then gives the name and location of the folder that will contain the individual ADOPS project files. The base configuration can be changed at this time; however, if none is specified the default configuration stored in the *‘system.conf’* file will be used. Finally, the user selects the FASTA files that will be used for the experiments and a new window is launched, showing the status (including the detection of positively selected amino acid sites) of each individual ADOPS project, each of which is named according to the names of FASTA files (Fig. 1). The name of the experiment of each individual project will be named “batch”.

## B+ Database

The B+ database is both a data repository and a convenient interface to browse the information contained in each ADOPS project interactively, without the need to download and unzip the corresponding ADOPS project. Thus, B+ allows the effortless exploration of the data contained in ADOPS projects.

**Fig. 2:**
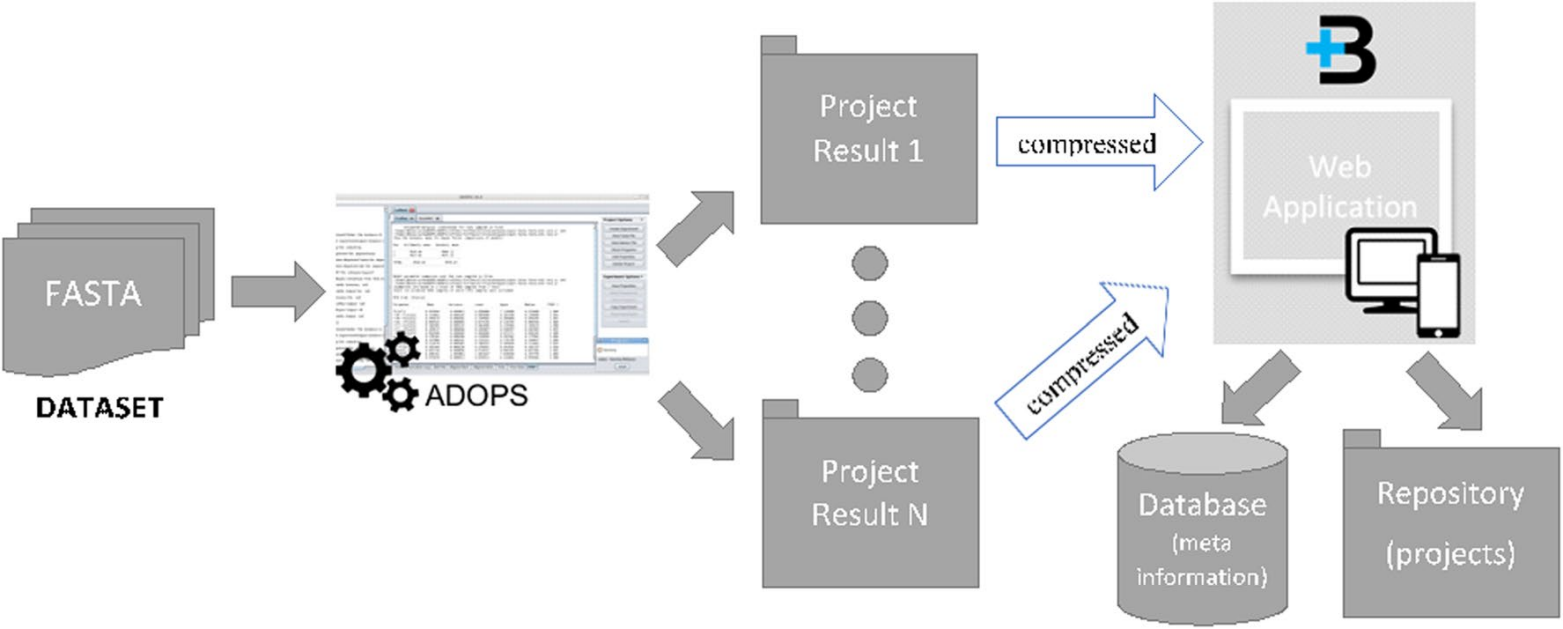
Graphical representation of the common workflow that researchers follow when performing large studies with ADOPS and B+

The common workflow when performing a study with ADOPS and B+ is summarized in Fig. 2. A researcher with a dataset to be studied, comprising several FASTA files, will use the ADOPS’ batch mode to analyze each FASTA file. On this mode, ADOPS will create a new project for each file, to analyze it. At the same time, the researcher will create a new dataset in B+ by simply providing a name and a description of the related study. Once the dataset is created, the ADOPS projects previously created can be uploaded even if they have not yet been fully analyzed, as they can be reloaded when completed. B+ will store the dataset and projects meta-information in a relational database, while the project files are stored in a local folder. Whenever appropriate, the researcher can make the dataset public, allowing any user to explore it.

B+ is open to any researcher wanting to share the result of large and small-scale analyses done with ADOPS, although contributing credentials are granted under request.

### Implementation

B+ was developed using the Laravel framework (https://laravel.com/) for web development and MySQL for database management. For a richer user interface, the Bootstrap framework (http://getbootstrap.com/) and the jQuery library (https://jquery.com/) were also used.

B+ repository is available at http://bpositive.i3s.up.pt/ and its source code is publicly available at https://github.com/sing-group/bpositive, under a GNU GPL 3.0 Open Source License (http://www.gnu.org/copyleft/gpl.html).

### Database Exploration

The B+ repository exploration interface is divided into three visualization levels: (1) dataset list level, introducing each dataset available under the platform; (2) dataset view, showing the projects which compose a dataset; and (3) the project view, presenting the different result views of a project. Thus, the visualization levels are arranged from the most general to the most detailed view of the stored data.

**Fig. 3:**
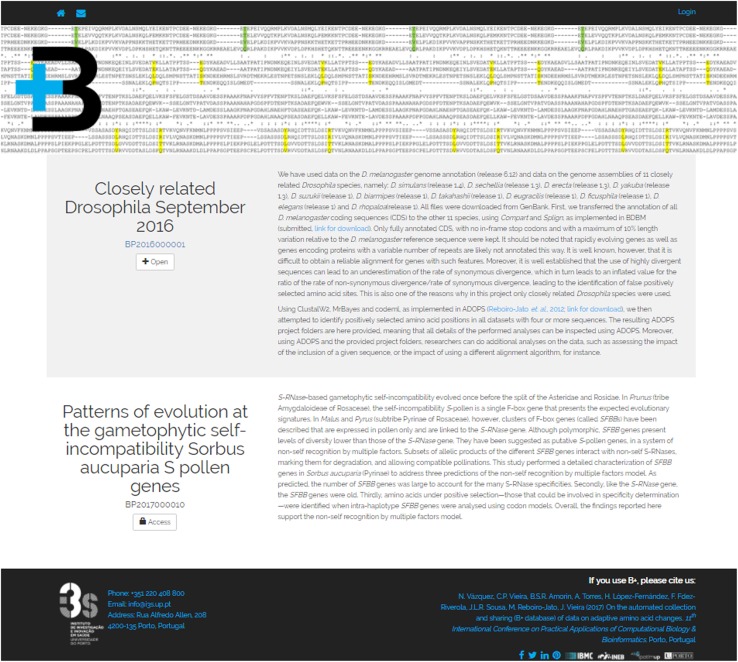
Dataset list view presenting several datasets

At the dataset list level (Fig. 3) users have access to all the datasets stored in the B+ repository. For each dataset, a title, a unique identifier and a brief description of the performed analysis are presented. In addition, “Open” or “Access” buttons are shown, depending on whether the dataset is public or private.

**Fig. 4:**
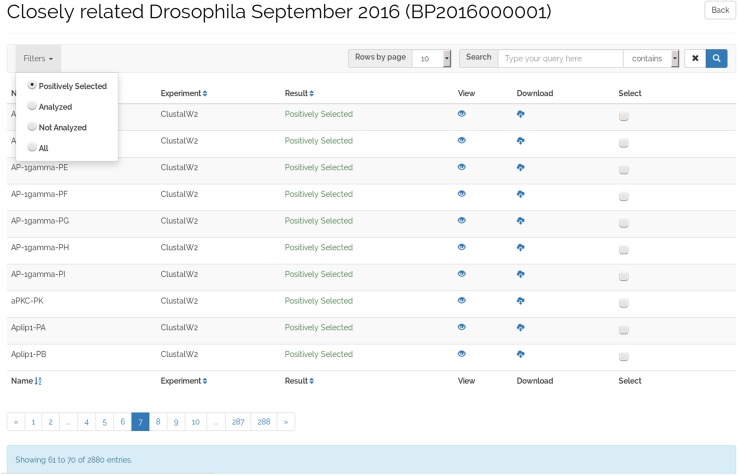
Dataset view presenting the ADOPS projects of a dataset

The dataset view (Fig. 4) allows user to explore the projects that compose a dataset. As the full analysis of a dataset can take several days (even months), B+ allows the storage of datasets partially analyzed, which enables users to have early access to the completed ADOPS projects results. Moreover, once a project is analyzed, the presence of positively selected amino acid sites can be confirmed or discarded. On this basis, any project in a dataset can be classified into one of three different states: (1) not analyzed, when the project has not yet been analyzed; (2) analyzed, when the project was analyzed but no positively selected amino acid sites were identified; or (3) positively selected, when the project was analyzed and positively selected amino acid sites were identified.

As seen in Fig. 4, the dataset view has a table arrangement of ten rows per page that can be changed using the “Number of entries” field. Several search options are provided at the top menu bar of the interface, and they can be executed in the database on the server side to provide maximum performance. The pagination is also handled on the server side to minimize the transfer of unnecessary data to the client. The free text search matches full and partial words using name and description fields of the database, also allowing the use of regular expressions.

The project view presents the results of an ADOPS project in the same way that the desktop application does. Each result is presented in a different tab, allowing users to explore them directly in the B+ web or to download them in the appropriate format.

**Fig. 5:**
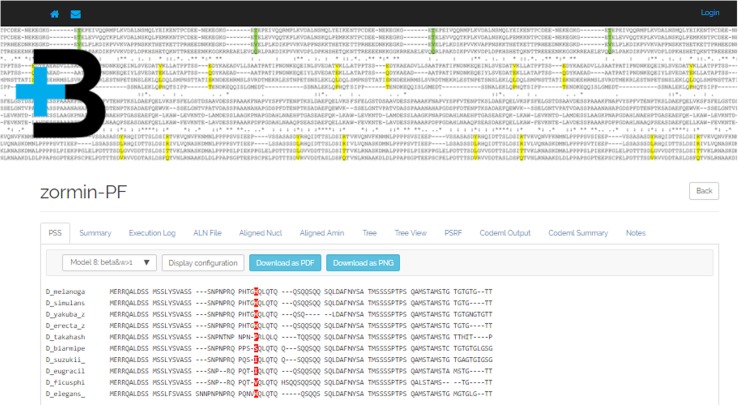
Project view presenting an ADOPS project with positively selected amino acid sites

As seen in Fig. 5, the first tab of the project view is a viewer for positively selected amino acid sites, which can be configured dynamically to match user preferences. It also allows downloading a PDF or PNG file with the result. Another tab that includes a viewer is the “Tree View”. Using the PhyD3 JavaScript library (https://phyd3.bits.vib.be/), this view shows an unrooted phylogram/cladogram for each tree available in the record. It can be also configured and the result can be downloaded in PNG or SVG formats. The remaining tabs present plain text documents.

### Management Interface

**Fig. 6:**
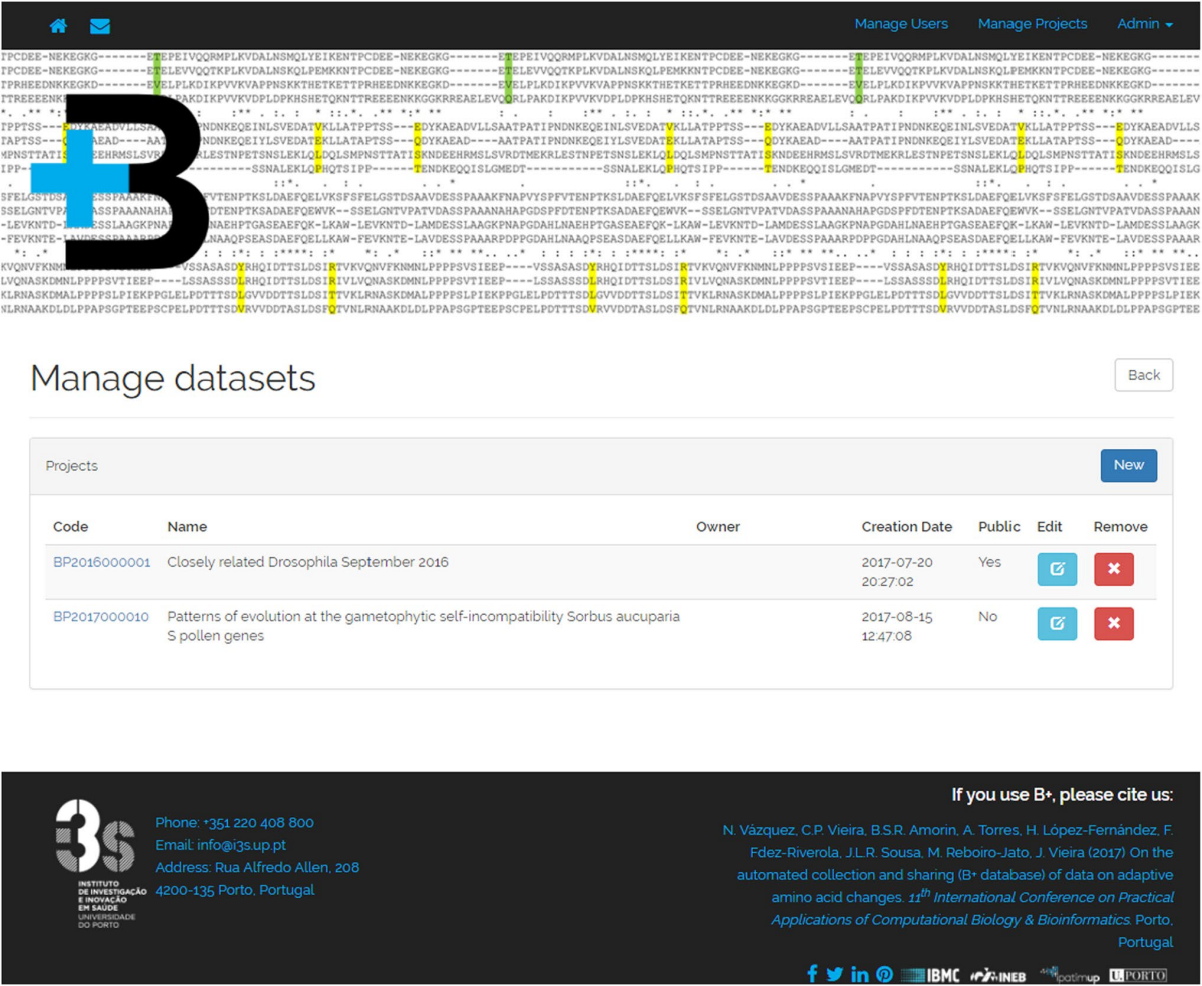
Manage datasets view

B+ also features a management interface, available only to users with specific privileges, to control users and datasets registered in the repository (Fig. 6). Specifically, B+ allows for two different privileged user profiles: (1) administrators, to manage all the users and datasets; and (2) contributors, to manage only their own datasets. Apart from managing the datasets metadata and the projects that compose it, dataset management also includes visibility control. In B+, projects can be public, which are visible by any user, or private, which can be viewed only by administrators, owner contributors, or those using a password set by administrators or contributors.

**Fig. 7:**
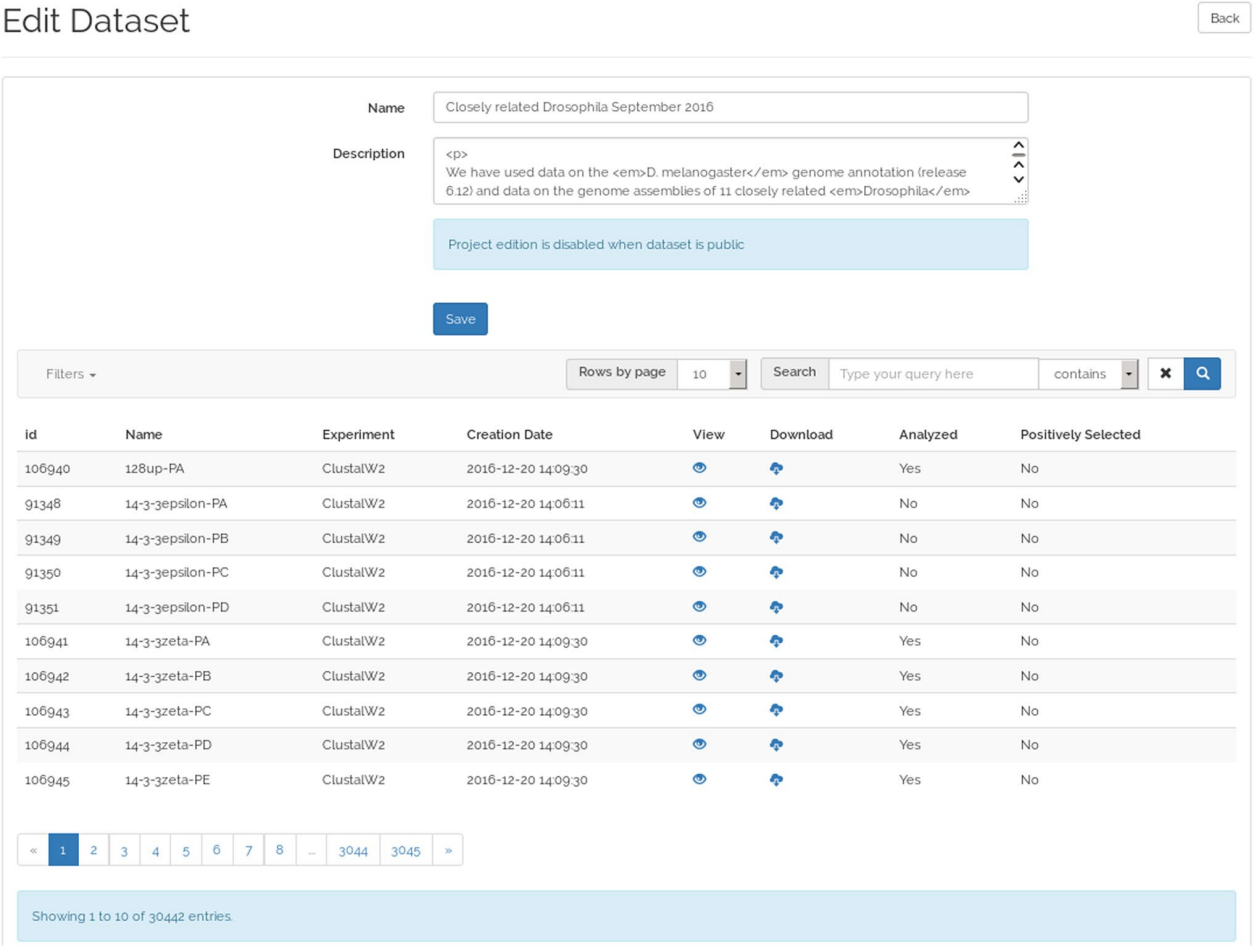
Edit dataset view

Every dataset uploaded to the repository is automatically checked to ensure that the ADOPS format is correct and that every result is displayed correctly. Datasets can be uploaded in bundle files including one folder for each ADOPS project, or one file for each project in a multiple upload. Allowable formats are “zip” and “tar.gz”. Once files are uploaded and validated, they are stored in the B+ repository and can be viewed immediately. Administrators can at any time edit the metadata and/or add, update or remove projects belonging to a dataset, while owner contributors can do the same when the project state is private only (Fig. 7). To ensure the stability of public data, after a project has been made public by the owner contributor, only administrators can make it private again.

## Usefulness

The first large scale dataset available at B+ is the “Closely related Drosophila dataset (2016)”. In brief, ADOPS projects for 19,652 Drosophila transcripts were generated (the details on how the sequence data were obtained and the analyses performed is provided at the B+ database under the project description), 14.6% of which show signs of positive selection (1200 genes), although human curated analyses must now be performed to validate these automatic inferences. These analyses are the first step toward the identification of the genes and amino acid sites that contribute to adaptation.

While ADOPS is intended to be a flexible and easy to use pipeline aimed at making robust inferences on positively selected amino acid sites, the information contained in the B+ database may serve many additional purposes. For instance, since a Bayesian phylogenetic tree is always generated and the corresponding NEWICK tree file saved, a robust tree for the relationship of the species analyzed could be easily created using applications such as CLANN [29], which would then allow for the construction of supertrees from partially overlapping species datasets. Moreover, ADOPS projects always provide the nucleotide and protein sequences in FASTA format (aligned and non-aligned), which can be used for many other types of analyses, including the identification of invariant (likely functionally important) amino acid sites. Moreover, the codeml tab provides information on the percentage of amino acid sites that are strongly conserved, neutral and adaptive. It should be noted that the “notes.txt” (the information shown in the notes tab) file under the folder with the name of the ADOPS experiment is a convenient way to store plain text results obtained with additional software applications, which may help the user with the interpretation of the data.

The ADOPS projects available at B+ can be downloaded, unzipped, and opened using the ADOPS GUI. When this is done, additional analyses can be performed, such as testing the impact of a different alignment algorithm on the results. Therefore, the availability of such a database ensures results repeatability, promotes data reuse with significant savings in the time needed to prepare datasets, and effortlessly allows further exploration of the data contained in ADOPS projects. In the new ADOPS version, there is also an option for adding new sequences to a given project, a tool that is certainly useful when the sequences that a given researcher needs are not all contained in the original ADOPS project.

## Conclusion

The ADOPS batch option allows running hundreds or even thousands of projects in a short period of time, without human intervention. B+ is both a data repository and a convenient interface to look at the information contained in ADOPS projects. The ADOPS projects can be downloaded, unzipped, and opened using the ADOPS GUI (https://www.sing-group.org/ADOPS/). Therefore, researchers can repeat the analyses, reuse the sequence and phylogenetic trees data, and make novel analyses without losing time on input file preparation. B+ currently holds a large dataset and several small datasets, although more will soon be available. Furthermore, the research community is welcome to contribute with other projects as well, even with small datasets. B+ will increase the repeatability of published analyses on the inference of positively selected amino acid sites, and make the process of reading articles more interactive.
